# Exploring the relationship between depression and delinquency: a sibling comparison design using the NLSY

**DOI:** 10.3389/fpsyg.2024.1430978

**Published:** 2024-07-01

**Authors:** Emma E. Sims, Jonathan D. Trattner, S. Mason Garrison

**Affiliations:** ^1^Department of Psychology, Wake Forest University, Winston Salem, NC, United States; ^2^Department of Interdisciplinary Studies, Wake Forest University, Winston Salem, NC, United States

**Keywords:** depression, criminal behavior, justice-involved youth, delinquency, sibling comparison

## Abstract

Relative to the general population, adolescents with psychiatric disorders such as major depression disorder are incarcerated (and reincarcerated) at higher rates. Current research is mixed on whether this association is a cause, consequence, or the product of selection. For example, aggression can lead to more depressive symptoms, yet depression is associated with antisocial behaviors (e.g., delinquency). To better understand the relationship between depression and delinquent behavior, we used the discordant kinship model to examine data from sibling pairs in the National Longitudinal Surveys of Youth 1979, a nationally representative study. By explicitly modeling within- and between-family variance, we reduced the influence of genetic and shared-environmental confounds on our analysis. Our results suggest that the relationship between depression and delinquency is not causal, but rather a product of selection.

## 1 Introduction

In the juvenile justice system, depression is the most commonly diagnosed mental illness, overrepresented relative to the general population (Fazel et al., 2008; Ozkan et al., 2019; Poyraz Fındık et al., 2019). This overrepresentation of depression is observed globally and persists throughout the lifespan (Maniadaki and Kakouros, 2008; Matsuura et al., 2009; Bebbington et al., 2017; Gaete et al., 2018; Forry et al., 2019). The causal link between criminal behavior and depression in adolescents remains uncertain, resulting in a variety of explanations. However, it is clear that these conditions often co-occur during adolescence (Fazel et al., 2008; Poyraz Fındık et al., 2019; Beaudry et al., 2020).

This paper aims to examine the causal relationship between depression and delinquency, specifically during adolescence, a stage characterized by a peak in criminal behavior (Ostrowsky and Messner, 2005; Reising et al., 2019). In the following sections, we review the existing literature for depression as a cause of delinquency and vice versa. We consider potential third variables, such as socioeconomic status, comorbid psychiatric illnesses, individual differences, and gender (Vaske et al., 2011; Defoe et al., 2013; Fontaine et al., 2019; Beaudry et al., 2020). Finally, we present our study conducted on a nationally-representative sample, within a genetically-informed framework.

## 2 Cause or confound?

Numerous explanations for the relationship between depression and delinquency have been proposed. These range from viewing depression as a consequence of delinquency (e.g., the Failure Model; Capaldi, 1992) to positing depression as a causal factor leading to later delinquency (e.g., General Strain Theory; Ganem, 2010). The third major theory, the Shared Risk Model, presents an equally viable alternative explanation, attributing the link between criminal behavior and mental illness to third variables like socioeconomic status, familial influences, or life stress (Wolff and Ollendick, 2006; Defoe et al., 2013; Walker et al., 2019).

Many studies inadvertently introduce confounding factors by relying on between-family designs (Rowe and Rodgers, 1997; Rodgers et al., 2000, 2001; Lahey and D’Onofrio, 2010; Garrison and Rodgers, 2016, 2019). These designs cannot discern processes generating differences between and among families, limiting conclusive support for the causal link (Kofler et al., 2011). Genetic and environmental influences may introduce confounds (Gjone and Stevenson, 1997; Sitnick et al., 2019). Neglecting these influences complicates accurate attribution of variance sources, potentially resulting in misattributions of causality (Rowe and Rodgers, 1997; Shadish et al., 2001; Rutter, 2007; Garrison et al., 2023). Therefore, it is critical to assess whether the relationship is causal or if it is primarily a theoretically-attractive confound. In this study, we address this concern by employing a genetically-informed sibling model design to control for confounds that introduce ambiguity in causality.

### 2.1 Depression as a cause

#### 2.1.1 General strain theory

According to General Strain Theory theory, strain induces negative emotions, and engaging in criminal activity serves as a means of alleviating these emotions (Agnew, 1992, 2006; Brezina, 1996; Ganem, 2010; Jang et al., 2016). Strain encompasses conditions disliked by individuals, which can manifest as loss of valued stimuli or the addition of negative stimuli. Criminal behavior is viewed as a coping mechanism for emotions like depression as it mitigates these feelings. The argument posits that individuals facing strain lack the resources to effectively manage it (Agnew, 1992, 2006; Brezina, 1996; Ganem, 2010; Jang et al., 2016). Specifically, depression tends to emerge when individuals attribute their adversity to themselves, leading to feelings of despair, and, consequently, delinquency (Agnew, 1992; Brezina, 1996; Kelly et al., 2019; Ozkan et al., 2019). Additionally, sociodemographic factors may also influence the propensity for experiencing negative emotions in response to strain (Ganem, 2010; Mirowsky and Ross, 2017). General Strain Theory constitutes one of many important models, including the Acting-Out Model, exploring depression as a causal factor.

#### 2.1.2 Acting-out model

The Acting-Out Model asserts that depressive symptoms may manifest behaviorally through criminal actions (Carlson and Cantwell, 1980; Gold et al., 1989; Akse et al., 2007). Numerous studies argue that depression serves as a precursor to delinquency, rather than the reverse (Akse et al., 2007; Kofler et al., 2011). For example, adolescent depression has been associated with an elevated likelihood of subsequent violent behavior (Ritakallio et al., 2006; Yu et al., 2017), as well as nonviolent offenses like vandalism (Ritakallio et al., 2006; Kelly et al., 2019). Early depressive symptoms better forecast future delinquent behavior changes than early delinquency does for subsequent depressive symptoms (Kofler et al., 2011). Within these frameworks, depression could be regarded as one of the contributors to criminal behavior.

### 2.2 Criminal behavior as a cause

The literature also suggests that criminal behavior can lead to depression. For example, aggressive behaviors have been linked to later depression (Kofler et al., 2011; Fanti et al., 2019a). One prominent model elucidating this relationship is the Failure Model.

#### 2.2.1 The failure model

The Failure Model proposes that early involvement in criminal behavior leads to failures in diverse domains, including social and academic contexts. These failures subsequently give rise to negative emotional responses and internalizing difficulties, encompassing symptoms of depression and anxiety (Patterson and Stoolmiller, 1991; Capaldi, 1992; Ward et al., 2010; Kofler et al., 2011). Children who encounter failure across multiple areas are more likely to face challenges in academic and social adjustment, rendering them more vulnerable to depressive symptoms (Capaldi and Stoolmiller, 1999). Longitudinal studies, such as Van der Giessen et al.’s (2013) multi-informant cross-lagged panel analysis, provide support for the Failure Model from early adolescence. Additionally, delinquency exhibits a robust association with an elevated risk of subsequent depressive disorder compared to national rates (Fanti et al., 2019a; Huesmann et al., 2019). The Failure Model finds support in evidence demonstrating that various forms of criminal behavior predict depression in adolescents, even when accounting for adverse life events.

### 2.3 Third variables as cause

The Shared Risk Model (Wolff and Ollendick, 2006) posits that the co-occurrence of delinquency and depression may be attributed to correlated third variables rather than a direct causal relationship (Beyers and Loeber, 2003; Wolff and Ollendick, 2006; Kofler et al., 2011). These third variables span a range of factors, including societal influences such as socioeconomic status (Defoe et al., 2013), individual differences (Lemos and Faísca, 2015; Barboza, 2020) like psychiatric comorbidities (Beaudry et al., 2020), and gender (Vaske et al., 2011).

Despite the breadth of these potential shared risk factors, their collective impact on the relationship between delinquency and depression remains under examined. Illustrating this complexity, a study by Jennings et al. (2019), on Hispanic youth found that depression was a significant differentiator for only the most chronic forms of offending, after accounting for multiple confounding variables, including self-esteem and stressful life events and potential selection effects (Jennings et al., 2019). While the specific third variables have yet to be conclusively identified, the Shared Risk Model remains a viable alternative explanation for the correlation between depression and delinquency (Wolff and Ollendick, 2006). For more comprehensive information, we recommend consulting the following reviews (Hoeve et al., 2009; Farrington et al., 2016; Basto-Pereira and Farrington, 2022).

#### 2.3.1 Gender differences

There are well-established gender differences for both depression and delinquency. Several studies have found that adolescent girls are more frequently diagnosed with major depression than their male counterparts (Weissman and Klerman, 1985; Fazel et al., 2008; Girgus and Yang, 2015; Hyde and Mezulis, 2020), while adolescent boys demonstrate a higher likelihood of engaging in delinquent behavior (Steffensmeier and Allan, 1996; De Coster, 2005; Kruttschnitt, 2013; Elkington et al., 2015; Kamaei and Abolhasani, 2020). Adolescent girls are more likely to have co-occurring delinquent behavior and depression (Wiesner and Kim, 2006; Elkington et al., 2015), and experience more rapid increases and slower decreases in depression symptoms compared to boys (Diamantopoulou et al., 2011). Conversely, among boys exhibiting high levels of delinquent behavior, this behavior predicts depressive symptoms more accurately than the reverse relationship (Beyers and Loeber, 2003; Wiesner and Kim, 2006). Several variables have been identified that affect the way gender influences the relationship. For instance, school functioning, caregiver strain, and social problems predict dual involvement in mental health services and the juvenile justice system for boys, whereas social problems, ethnicity (specifically African American), caregiver strain, and school functioning serve as predictive factors for girls (Graves et al., 2007). Consequently, understanding the role of gender is essential when examining the association between depression and delinquency.

#### 2.3.2 Prior within-family analyses

Numerous studies have investigated depression and delinquency through a genetically-informed lens, revealing substantial familial components for both variables at the individual level. For depression, a shared-environmental component is commonly found (Burt, 2009; Polderman et al., 2015), while a genetic component is identified depending on the operationalization and population (Sullivan et al., 2000; Corfield et al., 2017; Cai et al., 2020; Baselmans et al., 2021). Similarly, delinquency and other criminal behavior measures exhibit genetic and shared-environmental factors that vary with age and operationalization^1^ (Rhee and Waldman, 2002; Tuvblad and Baker, 2011). For example, a twin-family study found that externalizing disorders, including delinquency, showed substantial heritability and family transmission (Hicks et al., 2004). Regardless of the operationalization, criminal behavior consistently exhibits a genetic component (Walters, 1992; Beaver et al., 2009; Ling et al., 2019). Indeed it seems that previous studies have found that delinquency and other measures of criminal behavior are, in part, correlated because of overlapping genetic sources (Button et al., 2004; Ferguson, 2010). Furthermore, familial variables continue to have an effect on childhood disruptive behavior disorders even after accounting for confounding influences which is unlikely due solely to either genetic or environmental influences (Bornovalova et al., 2013).

Several studies have explored the association between depression and criminal behaviors and found overlapping genetic sources. For example, Rowe et al. (2008) found that genes contributed to the connection between oppositionality, physical aggression, delinquency, and antisocial traits with depression. They also observed phenotypic overlaps between antisocial behavior and depression, suggesting genetic, non-shared environmental, and trait-specific effects. Other studies have found that the covariation between depressive symptoms and antisocial behavior is genetically mediated, with both depression and antisocial behavior displaying individual genetic influences (Gjone and Stevenson, 1997; O’Connor et al., 1998). The stability of both antisocial and depressive symptoms was mainly accounted for by genetic factors for both cross-sectional and longitudinal genetic analyses.

In Sweden, two studies examined the association between depression and crime, using outpatient depression diagnoses in both twin and individual-level data (Fazel et al., 2015). Depressive symptoms were found to be associated with an increased risk of crime, even after adjusting for genetic and early environmental factors. The odds of violent crime in half-siblings and full siblings were significantly increased, demonstrating some familial confounding of the association between depression and violence. However, the odds increase remained significant in individuals with depression after adjusting for familial confounding and in those without substance abuse comorbidity or a previous violent conviction (Fazel et al., 2015).

Several studies have focused on genetic-based designs involving gender differences in depression and delinquency. Genetic risk factors for major depression contribute to three broad pathways (internalizing symptoms, externalizing symptoms, and psychosocial adversity) contributing to the risk for major depression in women (Kendler et al., 2002). For example, females in the highest genetic risk category had the same odds of offending as males in the low genetic risk group, suggesting that females might be less sensitive to the effects of genetic risk relative to males (Vaske et al., 2011). Several genetic-focused studies examine gender differences in the relationship between delinquency and depression.

## 3 Current study

The current literature presents mixed findings on the direction and causality of the relationship between depression and delinquency. Moreover, most studies have not considered the potential influence of genetic and environmental factors. Various theories, such as the General Strain Theory, Acting-Out Model, Failure Model, and Shared Risk Model, propose different explanations for this relationship. Further, existing research indicates gender differences in this context.

The current study adds to the literature by examining the relationship between depression and delinquency through a genetically-informed lens. Specifically, we compared siblings from a nationally representative sample, the NLSY. Our analysis focused on two main aspects: (1) determining if the relationship between depression and crime is present in both between- and within-family analyses while separating sources of variance, and (2) investigating any gender-specific effects in this relationship. We made the following predictions:

1. A positive correlation between depression and crime will emerge when accounting for different sources of variance.
2. The depression-delinquency relationship will be more pronounced in females than in males.
3. In adolescents, higher levels of depression will be associated with a higher likelihood of engaging in delinquent behavior.

## 4 Methods

### 4.1 Research design and analytic approach

We implemented a genetically-informed quasi-experimental design by leveraging sibling dyads. This design incorporates genetic and environmental elements, allowing for a powerful control of background variance associated with these differences (Rutter, 2007; Lahey and D’Onofrio, 2010). Specifically, we employed the discordant kinship model (Garrison and Rodgers, 2016; Garrison et al., 2023), a pooled-regression^2^ approach adapted from the reciprocal standard dyad model (Kenny et al., 2006).

As described by Garrison and Rodgers (2016) and Garrison et al. (2023), we compared individuals from within the family using the following models. First, we predict the difference in illegal behavior, $Y_{i\Delta }$ , for a given pair, indexed as i, in the following model:


$$Y_{i\Delta }=\beta_{0}+\beta_{1}{\bar{Y}}_{i}+\beta_{2}{\bar{X}}_{i}+\beta_{3}X_{i\Delta }+e_{i}$$

where


$$Y_{i\Delta }=Y_{i1}-Y_{i2};X_{i\Delta }=X_{i1}-X_{i2}$$

(Note that subscripts 1 and 2 identify the two individuals within the pair and are defined by $Y_{1}$ > $Y_{2}$ . Ties are randomly assigned.)

In this model, the relative difference in sibling outcome ( $Y_{i\Delta }$ ; e.g., delinquency) is predicted from the mean level of the outcome ( ${\bar{Y}}_{i}$ ; e.g., delinquency), the mean level of the predictor ( ${\bar{X}}_{i}$ ; e.g., depression), and the between-kin differences in the predictor ( $X_{i\Delta }$ ; depression). The mean levels, reflecting between-family variance, support causal inference through at least partial control for genes and shared environment in previous generations. Within this model, there is an explicit separation of within-family variance (with $Y_{i\Delta }$ and $X_{i\Delta }$ ) and between-family variance (with ${\bar{Y}}_{i}$ ; and ${\bar{X}}_{i}$ ), which allows for clear interpretation.

Thus, this model explicitly separates within-family variance and between-family variance, allowing for thus untangling between- and within-family influences. If a causal link exists between depression and delinquency, we expect kin differences in depression to be significantly associated with kin differences in crime. If the effect is not directly causal, we would expect no significant relationship between the differences in the illegal behavior outcome and the differences in the predictor, depression (see Table 1).

**Table 1:** Theory summaries.

Name	Causal	Theory	
General strain theory	Yes	Experiencing strain or stressors leads individuals to engage in delinquency as a way to cope or respond.	Agnew (1992, 2006), Brezina (1996), Ganem (2010), Jang et al. (2016)
Acting-out model	Yes	Adolescents with depression may engage in delinquency as a way to express their negative emotions or seek attention.	Wolff and Ollendick (2006), Akse et al. (2007), Kofler et al. (2011), Martínez-Ferrer and Stattin (2017), Ozkan et al. (2019)
Failure model	Yes	Early criminal behavior leads to failure which leads to depression	Van der Giessen et al. (2013)
Shared risk model	No	Common risk factors, such as family dysfunction or neighborhood disadvantage, contribute to both depression and delinquency.	Beyers and Loeber (2003), Wolff and Ollendick (2006), Kofler et al. (2011)

### 4.2 Sample

As described in Garrison and Rodgers (2016, 2019), the National Longitudinal Survey of Youth (NLSY79) is based on a nationally representative household probability sample sponsored in part by the U.S. Bureau of Labor Statistics. On December 31, 1978, 12,686 adolescents were identified within a household probability sample from 8,770 households. The initial sample consisted of three subsamples (a cross-sectional probability sample, an oversample of racial minorities and disadvantaged whites, and a military sample). We reviewed the civilian samples below in greater detail, as they were the only subsamples with siblings.

The two civilian samples consisted of a cross-sectional household probability sample of 6,111 non-institutionalized adolescents residing in the United States on December 31st, 1978, and a separate over-sampled civilian subsample of 5,295 racial minorities^3^ and “disadvantaged whites.” Subjects had birth dates ranging from January 1, 1957, to December 31, 1964, and were between 14 and 21 (mean age 17.90). Participants were surveyed annually until 1994 and have been surveyed biennially since. After the 1990 interview, all 1,643 “disadvantaged whites” from the oversample were deliberately dropped. Basic descriptive statistics are presented in Table 2. Throughout this paper, race has been dichotomized into a minority status variable, to be consistent with the sampling procedures implemented in 1978 (Ward et al., 2010), rather than the more intersectional approach (Cho et al., 2013). More information about the sampling process and the data can be found on the BLS website: http://www.bls.gov/nls/nlsy79.htm.

**Table 2:** Descriptive statistics by gender.

Characteristic	Mean	SD	N	Min	Max
Age	17.89	2.31	12,686	14	22
Delinquency	–	–	–	–	–
Delinquency (Total)	0.29	0.48	11,825	0	5.63
Delinquency (Female)	0.17	0.29	5,911	0	3.53
Delinquency (Male)	0.42	0.59	5,914	0	5.63
Depression	–	–	–	–	–
CESD 20 (Total)	9.75	9.29	8,978	0	59
CESD 20 (Female)	10.62	9.85	4,526	0	59
CESD 20 (Male)	8.87	8.59	4,452	0	56

#### 4.2.1 Sibling dyads

Using the R packages Nlsylinks (Beasley et al., 2023) and discord (Garrison et al., 2020), we created sibling dyads. The kinship links were identified using both indirect and direct ascertainment of relatedness, as part of a multi-year project (Rodgers et al., 2016), resulting in reliable and valid kinship classifications (e.g., twins, full siblings, half-siblings, etc.) for approximately 95% of the NLSY79 kinship pairs. For ease of interpretation, we restricted our sample to full siblings, and in families with more than two siblings, we selected the oldest two who had data.

Fully reproducible source code for these analyses are available at https://github.com/R-Computing-Lab/Sims-et-al-2024.

### 4.3 Measures

#### 4.3.1 Depression

The present study utilized the Center for Epidemiologic Studies Depression scale (CES-D) to measure depressive symptoms (Radloff, 1977). The NLSY employed two versions of the CES-D: a 20-item long form (collected in 1992) and a 7-item short form (collected in 1992 and 1994), both of which have been validated for their psychometric properties (Levine, 2013). Respondents were asked to endorse items, such as “I had a crying spell,” on a 4-point Likert scale, which assesses the frequency of each symptom experienced within the past week. The scale ranges from 0 (rarely or none of the time, less than 1 day) to 3 (most or all of the time, 5–7 days).

The long-form CES-D score is calculated by summing the responses to all 20 items, resulting in a possible range of 0 (no symptoms) to 60 (maximum symptoms; Przepiorka et al., 2019). For context, a score of 16 points or higher indicates depression (Hann et al., 1999). In contrast, the short form comprises seven items and yields scores from 0 to 21, with scores of 8 or higher signifying high risk for depression (Levine, 2013). If the short form scores 8 or more, they are considered depressed. In our sample, women had an average long-form CES-D score of 10.62 (SD = 9.85, *n* = 4,526), while men had an average score of 8.87 (SD = 8.59, *n* = 4,452; Table 2).

#### 4.3.2 Criminal behavior

The NLSY includes a variety of criminal behavior measures. Because we were interested in early criminal behavior, we focused on items collected in 1980 that were part of the “Delinquency scales.” This scale comprised 20 data-driven binary items that captured a range of delinquent activities, such as marijuana use (most frequent) and gambling (least frequent). To ensure the reliability of the analysis and prevent artificially deflating scores due to missing data, the average delinquent activity score for each participant was calculated.

In the NLSY sample, women reported an average delinquent activity score of 0.17 (SD = 0.29, n = 5,911), while men had an average score of 0.42 (SD = 0.59, *n* = 5,914). Detailed information about the Delinquency scales and item-level summary statistics can be found in the Bureau of Labor Statistics (BLS) documentation.^4^

## 5 Results

The current study examines the relationship between depression and delinquent behavior within a sibling comparison framework. Specifically, we examined these relationships in three stages: a classical regression model, a between-family model, and discordant kinship model. Throughout this process, we also considered the influence of gender, race, and age on these associations.

We evaluated the results for all the CES-D measures, but our primary focus was on the CES-D 20 as our depression measure, due to its inclusion of more items (20) compared to the alternative short-form versions. For readers interested in replicated analyses of the other two measures, please refer to the Appendix for supplemental analyses. We have noted any discrepancies between the results in the main text.

### 5.1 Individual level results

We began by summarizing the data without accounting for family structure. At this stage, we adopted a bidirectional approach to the OLS regression models, considering both depression and delinquency as outcome variables in separate analyses. When average delinquency score was the dependent variable there was a significant contribution from the predictors of depression, gender, age, and minority status yielding a significant F-statistic (201.57, df_1_ = 4, df_2_ = 8,493) and an adjusted R^2^ of 0.09 (see Table 3).

**Table 3:** Individual level results: CESD 20 depression predicts delinquency.

	Standardized beta	Beta	*t*-statistic	95% CI^a^	*p*-value
Intercept	−0.318	0.444	11.1	0.365, 0.522	<0.001
CESD 20	0.102	0.005	9.72	0.004, 0.006	<0.001
Gender					
Female	–	–	–	–	–
Male	0.526	0.249	25.2	0.230, 0.269	<0.001
Age	−0.094	−0.020	−9.05	−0.024, −0.016	<0.001
Ethnicity					
Minority	–	–	–	–	–
Non-minority	0.115	0.054	5.48	0.035, 0.074	<0.001

R^2^ = 0.087; F-Statistic = 202; DF1 = 4; DF2 = 8,493; *p*-value = <0.001; No. Obs. = 8,498.

a CI, confidence interval.

Subsequently, we reversed the direction of analysis, designating the CES-D 20 score as the outcome variable while using delinquency, gender, age, and minority status as independent variables. This model also yielded a significant F-statistic (83.68, df_1_ = 4, df_2_ = 8,493) and an adjusted R^2^ of 0.04 (see Table 4).

**Table 4:** Individual level results: Delinquency predicts CESD 20 depression.

	Standardized beta	Beta	*t*-statistic	95% CI^a^	*p*-value
Intercept	0.261	11.1	13.9	9.55, 12.7	<0.001
Delinquency	0.108	2.10	9.72	1.68, 2.53	<0.001
Gender					
Female	–	–	–	–	–
Male	−0.239	−2.21	−10.9	−2.61, −1.81	<0.001
Age	0.005	0.022	0.496	−0.064, 0.108	0.620
Ethnicity					
Minority	–	–	–	–	–
Non-minority	−0.283	−2.61	−13.3	−3.00, −2.23	<0.001

R^2^ = 0.038; F-Statistic = 83.7; DF1 = 4; DF2 = 8,493; *p*-value = <0.001; No. Obs. = 8,498.

a CI, confidence interval.

Without accounting for family structure, depression (*β* = 0.102), gender (*β* = 0.526), and minority status (*β* = 0.115) significantly predicted delinquency levels. A unit increase in the depression score corresponded to a 0.102 standard deviation increase in delinquency. Males had a 0.526 standard deviation higher delinquency score compared to females. Non-Black non-Hispanic individuals had a delinquency score that was 0.115 standard deviations higher than other groups. Older age also significantly predicted lower delinquency levels (*β* = −0.094).

In the alternative analysis, CES-D 20 score was the dependent variable and average delinquency, gender, age in 1979, and minority status served as predictors. The standardized coefficients indicated that delinquency (*β* = 0.108), gender (*β* = −0.239), and minority status (*β* = −0.283) were significant predictors of depression level. Each unit increase in average delinquency corresponded to a 0.108 standard deviation increase in the depression score. Males, on average, had a 0.239 standard deviation lower depression score compared to females. Non-Black non-Hispanic individuals had a depression score that was 0.283 standard deviations lower than other groups. Age was not a significant predictor.

### 5.2 Between-family results

We next examined the between-family results, focusing on the association between sibling averages in depression and delinquency. We tested this relationship in both directions, investigating whether delinquency predicted depression, and whether depression predicted delinquency. As expected, the results were similar for both directions.

#### 5.2.1 Depression predicting delinquency

Sibling averages of depression were used to predict sibling averages of delinquency. Table 5 presents the unstandardized regression results (*n* = 2,957). The F-statistic (51.38, df_1_ = 5, df_2_ = 2,951) was significant, and the adjusted R^2^ was 0.08. A standard deviation increase in the average standardized depression predicted a statistically significant increase of 0.0136 standard deviations in average delinquency. As expected, gender composition, age, and minority status were also predictive of criminal activity. Relative to the reference group (of respondents classified as either Black or Hispanic), siblings from non-Black non-Hispanic families had higher family levels of delinquency (*β* = 0.224). Older sibling pairs were predictive of lower levels of delinquency (*β* = −1.06). Relative to the reference group of two brothers, families with more sisters had lower levels of delinquency (one sister *β* = −0.322; two sisters *β* = −0.604).

**Table 5:** Between family results: CESD 20 depression predicts delinquency.

	Standardized beta	Beta	*t*-statistic	95% CI^a^	*p*-value
Intercept	0.196	0.758	9.88	0.608, 0.908	<0.001
CESD 20 Mean	0.136	0.008	7.56	0.006, 0.010	<0.001
Gender					
Male siblings	–	–	–	–	–
Female siblings	−0.604	−0.222	−12.3	−0.257, −0.186	<0.001
Mixed gender siblings	−0.322	−0.118	−7.59	−0.149, −0.088	<0.001
Age	−0.106	−0.026	−5.98	−0.035, −0.017	<0.001
Ethnicity					
Minority	–	–	–	–	–
Non-minority	0.224	0.082	6.22	0.056, 0.108	<0.001

R^2^ = 0.080; F-Statistic = 51.4; DF1 = 5; DF2 = 2,951; p-value = <0.001; No. Obs. = 2,957.

a CI, confidence interval.

#### 5.2.2 Delinquency predicting depression

Sibling averages of delinquency were used to predict sibling averages of depression. Table 6 presents the unstandardized regression results (*n* = 2,957). The F-statistic (37.90, df_1_ = 5, df_2_ = 2,951) was significant, and the adjusted R^2^ was 0.06. A standard deviation increase in the average standardized delinquency predicted a statistically significant increase of 0.139 standard deviations in average depression. As expected, gender composition and minority status were also predictive, whereas age was not. Relative to the reference group, non-Black non-Hispanic families had higher family levels of depression (*β* = −0.415). Relative to the reference group of brothers, families with sisters had higher averages in depression (*β* = 0.263). For the between family results, delinquency predicted siblings’ average depression score, as well as the other way around.

**Table 6:** Between family results: Delinquency predicts CESD 20 depression.

	Standardized beta	Beta	*t*-statistic	95% CI^a^	*p*-value
Intercept	0.080	9.02	6.41	6.26, 11.8	<0.001
Delinquency Mean	0.139	2.50	7.56	1.86, 3.15	<0.001
Gender					
Male siblings	–	–	–	–	–
Female siblings	0.263	1.74	5.18	1.08, 2.39	<0.001
Mixed gender siblings	0.117	0.769	2.70	0.210, 1.33	0.007
Age	0.001	0.006	0.075	−0.150, 0.162	0.940
Ethnicity					
Minority	–	–	–	–	–
Non-minority	−0.415	−2.74	−11.6	−3.20, −2.27	<0.001

R^2^ = 0.060; F-Statistic = 37.9; DF1 = 5; DF2 = 2,951; *p*-value = <0.001; No. Obs. = 2,957.

a CI, confidence interval.

### 5.3 Joint within and between family results

Finally, we jointly examined within-family and between-family effects, using the discordant kinship model. In this pool-regression approach, sibling differences in the outcome are predicted from sibling differences in several covariates, while also controlling for between-family covariates. This method allows us to examine the within-family relationships. We modeled the relationship between sibling differences in depression and differences in delinquency in both directions while controlling for the means of outcomes and predictors.

#### 5.3.1 Depression predicting delinquency

Sibling differences in depression were used to predict differences in delinquency, controlling for family-level gender composition, family minority status, and sibling averages of both delinquency and depression. Table 7 presents the unstandardized regression results (*n* = 2,957). The F-statistic (581.82, df_1_ = 8, df_2_ = 2,948) was significant, and the adjusted R^2^ was 0.61. A standard-deviation increase in the siblings’ average delinquency predicted a statistically significant increase of 0.785 standard deviations in the average of the siblings’ depression score. Neither sibling differences in depression were significantly associated with sibling differences in criminal behavior. Age and gender were significant factors. The wider the age gap between siblings, the narrower the difference in their depression scores (*β* = −0.030). Relative to the reference group, opposite sex siblings had greater differences in depression (*β* = 0.141).

**Table 7:** Discordant results: Differences in CESD 20 depression predicts differences in delinquency.

	Standardized beta	Beta	*t*-statistic	95% CI^a^	*p*-value
Intercept	−0.069	−0.067	−1.01	−0.197, 0.063	0.311
Delinquency Mean	0.785	1.03	65.6	0.995, 1.06	<0.001
CESD 20 Difference	0.004	0.000	0.376	−0.001, 0.001	0.707
CESD 20 Mean	−0.004	0.000	−0.376	−0.002, 0.001	0.707
Age Difference	−0.030	−0.005	−2.60	−0.009, −0.001	0.009
Age Mean	0.014	0.005	1.21	−0.003, 0.012	0.225
Gender					
Male siblings		—	—	—	—
Female siblings	0.028	0.013	0.854	−0.017, 0.044	0.393
Mixed gender siblings	0.141	0.068	5.07	0.042, 0.094	<0.001
Ethnicity					
Minority		–	–	–	–
Non-minority	−0.012	−0.006	−0.516	−0.028, 0.016	0.606

R^2^ = 0.612; F-Statistic = 582; DF1 = 8; DF2 = 2,948; *p*-value = <0.001; No. Obs. = 2,957.

a CI, confidence interval.

#### 5.3.2 Delinquency predicting depression

Sibling differences in delinquency were used to predict differences in depression. Table 8 presents the unstandardized regression results (*n* = 2,957). The F-statistic (222.07, df_1_ = 8, df_2_ = 2,948) was significant, and the adjusted R^2^ was 0.37. A standard-deviation increase in the siblings’ average depression predicted a statistically significant increase of 0.622 standard deviations in the average of the siblings’ delinquency score. A standard-deviation increase in the siblings’ age difference predicted a statistically significant decrease of 0.037 standard deviations in the difference of the siblings’ depression score. Relative to the reference group, siblings from non-Black non-Hispanic families had wider differences in depression scores (*β* = 0.120). Once you account for age, the results are consistent in that they suggest it is not a causal relationship in either direction.

**Table 8:** Discordant results: Differences in delinquency predicts differences in CESD 20 depression.

	Standardized beta	Beta	*t*-statistic	95% CI^a^	*p*-value
Intercept	−0.064	0.535	0.382	−2.21, 3.28	0.703
Delinquency Mean	−0.003	−0.056	−0.168	−0.708, 0.597	0.867
Delinquency Difference	0.009	0.114	0.580	−0.271, 0.499	0.562
CESD 20 Mean	0.622	0.754	41.4	0.719, 0.790	<0.001
Age Difference	−0.037	−0.102	−2.51	−0.182, −0.022	0.012
Age Mean	0.004	0.020	0.252	−0.134, 0.174	0.801
Gender					
Male siblings	–	–	–	–	–
Female siblings	−0.002	−0.018	−0.053	−0.671, 0.635	0.958
Mixed gender siblings	0.013	0.105	0.370	−0.450, 0.659	0.711
Ethnicity					
Minority	–	–	–	–	–
Non-minority	0.120	0.962	4.02	0.493, 1.43	<0.001

R^2^ = 0.376; F-Statistic = 222; DF1 = 8; DF2 = 2,948; *p*-value = <0.001; No. Obs. = 2,957.

a CI, confidence interval.

## 6 Discussion

This research examined the association between depression and delinquency using multiple regression analyses at the individual, between-, and within-family levels. At the individual level, significant associations between depression and delinquency corroborate existing psychological theories such as the Failure model (Capaldi, 1992) and the General Strain Theory (Agnew, 1992). However, subsequent analyses at the between-family and within-family levels reveal a more nuanced picture. Significant between-family effects in both directions, suggest that shared familial factors such as genetic and environmental influences may drive the observed relationships, while the absence of significant within-family effects suggests that the relationship is not directly causal but rather driven by those shared familial factors. In other words, this pattern of results is consistent with the Shared Risk Model (Wolff and Ollendick, 2006; Defoe et al., 2013; Walker et al., 2019). These findings emphasize the importance of considering both individual and family contexts in understanding the etiology of delinquency and depression, challenging simplistic causal interpretations and underscoring the need for a multi-layered analytical approach in psychological research. Further, these results indicate that interventions should target those broader familial contexts rather than focusing solely on individual-level factors.

### 6.1 Individual level results

Our findings, without accounting for family structure, corroborate previous research that associates increased delinquency in adolescents with higher depression levels (Kofler et al., 2011; Van der Giessen et al., 2013). These outcomes support theories such as the Acting-Out model, which suggests that symptoms of depression manifest through delinquent behavior, and the General Strain Theory, which posits that strain leads to negative emotions and consequently, to delinquency (Agnew, 1992, 2006; Ganem, 2010). Likewise, gender effects aligned with previous research, showing that boys are more likely to engage in criminal activities (Jang et al., 2016). Therefore, gender and increased depression appear to directly influence criminal activity. Additionally, sociodemographic variables like age, gender, race, socioeconomic status, marital status, and parental status may impact the likelihood of negative emotions in response to strain, particularly for those with lower socio-demographic status, leading to feelings of powerlessness (Ganem, 2010; Mirowsky and Ross, 2017).

### 6.2 Between family results

Our between-family analyses revealed an association between depression and crime, consistent with the individual level results. When examining delinquency as the outcome, we found that gender, age, minority status, and depression were significant predictors of criminal activity, aligning with the Shared Risk Model (Wolff and Ollendick, 2006; Defoe et al., 2013; Walker et al., 2019), replicating studies by Brezina (1996) and Taşkıran et al. (2017). Being in a minority group was not associated with higher levels of crime activity in our study. Furthermore, depression and gender positively predicted crime at the family level; families with male siblings had a higher average crime rate compared to families with female siblings, in line with previous studies indicating males are more likely to engage in criminal behavior under stressors (De Coster, 2005). Variables like home environment and emotion regulation distinguished violent offending boys from those without criminal records (Sitnick et al., 2019), indicating a direct influence of gender and increased depression on criminal activity. When examining depression as the outcome, delinquency, gender, and minority status predicted higher average depression scores among siblings, replicating past studies (Fazel et al., 2008; Fanti et al., 2019b). Notably, families with female siblings and non-minority families had higher averages in depression (Teplin et al., 2002; Walker et al., 2019). Delinquency predicted siblings’ average depression score, and vice versa.

### 6.3 Discordant kinship model results

At the within-family level, our study found no significant association between depression and delinquency among siblings, challenging prior research that suggests a direct link between these factors in youth (Yu et al., 2017). Instead, the relationship between depression and delinquency may be influenced by factors such as institutionalized poverty, personality traits, or intergenerational stress. Family-level interventions targeting personality change, good habits, or educational attainment could potentially impact this relationship, aligning with the Shared Risk Model (Wolff and Ollendick, 2006).

We identified several potential between-family third variables, such as socioeconomic status, family factors, or parental criminal behavior, which might explain the observed non-causal relationship between delinquency and depression (Walker et al., 2019). Factors like lack of energy and inactivity may also decouple depression from delinquent behaviors (Sigfusdottir et al., 2004). Controlling for various covariates, including socio-demographic background and mental health disorders, can diminish the significance of the causal model linking offending and later depression (Walker et al., 2019). Some studies also suggest that the consequences of criminal behavior, such as incarceration, are linked to depression rather than the behavior itself (Turney et al., 2012). Overall, various third variables could be contributing to the relationship between delinquency and depression.

Within-family analyses highlighted that differences in depression and delinquency were more pronounced in opposite-gender siblings, indicating the influence of a third variable regardless of the direction. Consistent with the age-crime curve (Shulman et al., 2013; Ulmer and Steffensmeier, 2014), age was a significant predictor, with wider age gaps between siblings associated with smaller differences in delinquency. For delinquency predicting depression, both minority status and age were significant predictors, with non-minority siblings showing higher levels of depression difference scores and wider age gaps leading to smaller differences in depression. Overall, when accounting for age, the results suggest that the depression-delinquency relationship is not causal in either direction.

Notably, our findings also showed that delinquency predicting depression exhibited no significant gender differences, consistent with other studies that found no gender differences in this relationship (O’Connor et al., 1998). However, these results contradict past studies indicating higher genetic risk for females compared to males (Vaske et al., 2011). These findings did not align with past research suggesting higher rates of major depressive disorder among females in the juvenile justice system (Teplin et al., 2002; Livanou et al., 2019; Beaudry et al., 2020). Genetic factors, specifically emotionality contributing to aggressive behaviors, may be influenced by gene–environment interactions, potentially explaining the absence of a direct causal relationship (Gjone and Stevenson, 1997). Significantly, the relationship between escalating depression and increasing delinquency trajectories was particularly evident in girls (Wiesner and Kim, 2006), underscoring a possible gender-specific pattern where childhood depression and delinquency predict membership in these trajectories (Diamantopoulou et al., 2011).

### 6.4 Limitations and future directions

This study, while providing valuable insights into the relationship between depression and delinquency, has several limitations that influence the interpretation of our findings and constrain the extent to which they can be generalized. We discuss these limitations in terms of their impact on internal and external validity.

### 6.5 Internal validity

#### 6.5.1 Self-report biases

Our study used the CES-D scale and delinquency scales for assessing depression and delinquency, respectively, both of which rely on self-reporting. Although these scales are widely used and validated (Radloff, 1977; Levine, 2013), they are not immune to potential biases. Social desirability bias may cause participants to underreport symptoms of depression or instances of delinquent behavior (Klassen et al., 1975; Naqvi and Kamal, 2013). Similarly, recall bias (Mathews and Bradle, 1983) could affect participants’ ability to accurately remember and report past feelings or behaviors (Kruijshaar et al., 2005; Kirk, 2006; Lynch and Addington, 2010; Gomes et al., 2019). To address these biases, behavioral measures or observer reports could be implemented to complement self-report measures.

#### 6.5.2 Sibling controls

Although the discordant kinship model is a powerful tool for examining within- and between-family variance, the study’s quasi-experimental nature limits the depth of our causal conclusions (See Garrison et al., 2023 for extensive discussion on this point). Our design is effective for eliminating causal claims, but is only partially effective at affirming causation, when full siblings are used (in contrast to monozygotic twins). Like most quasi-experimental designs, this design does not manipulate variables directly; rather, it relies on sibling comparisons to partially control genetic effects. Although unlikely, it does not fully eliminate the potential influence of genetic factors.

#### 6.5.3 Temporal ordering

Moreover, the temporal ordering of the delinquency and depression measures in the NLSY dataset presents a substantial challenge. Delinquency was assessed in 1980, well before the depression measures were collected in the early 1990s. This considerable time lag restricts our ability to definitively explore the directionality of these relationships. Alternative, genetically-informed designs can be used (e.g., Cholesky models, Heath et al., 1993) when the time gap is narrower. Furthermore, it is still possible that on a shorter timescale the relationships might be causal, but have dissipated with time. Our study design, while controlling for familial confounding, does not allow us to fully eliminate this threat to validity, highlighting the need for future research employing genetically-informed longitudinal designs with more repeated assessments across shorter timespans.

### 6.6 External validity

Using archival data introduces constraints, particularly in controlling for variables or biases. Our study uses the National Longitudinal Survey of Youth, one of the few nationally-representative genetic studies (Holden et al., 2022). While this sample is a rich and valuable dataset for exploring our research question, it limits the generalizability of our findings, particularly in different cultural contexts. The behaviors and experiences of American youth, represented in this sample, may not reflect those of youth in other countries or cultural contexts. Therefore, caution should be exercised when extrapolating these findings to other populations, especially to non-WEIRD samples (Ghai, 2021). Instead, we encourage replication of these findings using family datasets from other countries, such as the China Family Panel Survey (e.g., Lyu and Garrison, 2022 for details on their kinship links).

## 7 Conclusion

The goal of this research was to evaluate the three major explanations for the relationship between delinquency and depression through a genetically informed lens. Using sibling comparison models, we found no causal relationship between crime and depression in either direction. Instead, the relationship was driven by shared familial factors. These results aligned with the Shared Risk Model (Wolff and Ollendick, 2006), suggesting that third variables contribute to the observed association between depression and criminal behavior. In light of this pattern of results, effective interventions should target the family level rather than individuals.

By adopting a multi-layered analytical approach and considering both individual and familial factors, researchers can develop more nuanced models of human behavior and inform the development of effective intervention strategies. Social and personality psychologists can use these frameworks to develop and refine theoretical models explaining the interplay between mental health and behavior. Understanding these shared risk factors can help psychologists identify vulnerable populations and develop targeted interventions to address both depression and delinquency simultaneously.

Future research should evaluate whether current interventions could be improved by targeting the family and should also consider expanding the scope of research to different cultural contexts to improve the generalizability of the findings. Employing more advanced genetically informed models, such as those using twins, could further untangle the nuances of these familial associations through gene-by-environment interactions. In summary, this study casts doubt on the widely accepted causal link between depression and delinquency, emphasizing instead the role of shared familial and other external factors, in support of the Shared Risk model. These insights contribute to a more comprehensive understanding of the interplay between genetic, familial, and environmental factors in adolescent behavior, which is crucial for developing effective mental health interventions and advancing the field of psychology.

## Data availability statement

The original contributions presented in the study are included in the article/Supplementary material, further inquiries can be directed to the corresponding author. Fully reproducible source code for these analyses are available at https://github.com/R-Computing-Lab/Sims-et-al-2024.

## Ethics statement

Ethical approval was not required for the study involving humans in accordance with the local legislation and institutional requirements. Written informed consent to participate in this study was not required from the participants or the participants' legal guardians/next of kin in accordance with the national legislation and the institutional requirements.

## Author contributions

ES: Conceptualization, Data curation, Formal analysis, Investigation, Methodology, Project administration, Resources, Validation, Writing – original draft, Writing – review & editing. JT: Data curation, Software, Writing – review & editing. SG: Conceptualization, Data curation, Formal analysis, Investigation, Methodology, Project administration, Resources, Software, Supervision, Validation, Writing – original draft, Writing – review & editing.

## Footnotes

^1^Operationalization of antisocial behavior, assessment method, zygosity determination method, and age accounted for significant differences in the genetic and environmental influences on antisocial behavior (Rhee and Waldman, 2002).

^2^There are alternative techniques to distinguish between within- and between-level effects, such as multilevel modeling or regression with clustered standard errors (Begg and Parides, 2003). However, the regression model described above has the advantage of parsimony and is sufficient for our research question.

^3^Race was operationalised by the original NLSY79 investigators based on a combination of self-identification, interviewer report, and inference from household reports (Ward et al., 2010). This method resulted in three groups: Hispanic, Black, and non-Black-non-Hispanic respondents.

^4^https://www.nlsinfo.org/content/cohorts/nlsy79/topical-guide/crime/crime-delinquency-arrest-records
