# Supplementary material for: Exploring the relationship between depression and delinquency: a sibling comparison design using the NLSY
Source: Front Psychol. 2024 Jul 1;15:1430978. doi: 10.3389/fpsyg.2024.1430978 (PMC11247016; doi:10.3389/fpsyg.2024.1430978)
Supplement: Supplementary file 1 [file Data_Sheet_1.docx]

**Appendix**

## Table 1A

Delinquency and Depression Mean by Age

| **Characteristic** | **Mean** | **SD** | **N** | **Min** | **Max** |
| --- | --- | --- | --- | --- | --- |
| 14 | - | - | - | - | - |
| Delinquency | 0.35 | 0.49 | 914 | 0 | 4.31 |
| CES-D 7 1992 | 4.24 | 3.89 | 767 | 0 | 21 |
| CES-D 20 | 9.72 | 8.94 | 769 | 0 | 48 |
| CES-D 7 1994 | 3.67 | 3.81 | 773 | 0 | 21 |
| 15 | - | - | - | - | - |
| Delinquency | 0.36 | 0.534 | 1501 | 0 | 5.63 |
| CES-D 7 1992 | 4.38 | 4.08 | 1237 | 0 | 21 |
| CES-D 20 | 10.23 | 9.38 | 1241 | 0 | 51 |
| CES-D 7 1994 | 3.83 | 4.12 | 1236 | 0 | 21 |
| 16 | - | - | - | - | - |
| Delinquency | 0.35 | 0.53 | 1481 | 0 | 5.31 |
| CES-D 7 1992 | 4.17 | 3.92 | 1244 | 0 | 21 |
| CES-D 20 | 9.61 | 9.06 | 1250 | 0 | 59 |
| CES-D 7 1994 | 3.75 | 3.95 | 1223 | 0 | 21 |
| 17 | - | - | - | - | - |
| Delinquency | 0.34 | 0.51 | 1431 | 0 | 4.33 |
| CES-D 7 1992 | 4.14 | 4.10 | 1227 | 0 | 21 |
| CES-D 20 | 9.48 | 9.43 | 1233 | 0 | 54 |
| CES-D 7 1994 | 3.70 | 4.05 | 1215 | 0 | 21 |
| 18 | - | - | - | - | - |
| Delinquency | 0.31 | 0.49 | 1509 | 0 | 4.53 |
| CES-D 7 1992 | 4.15 | 4.06 | 1232 | 0 | 21 |
| CES-D 20 | 9.68 | 9.41 | 1240 | 0 | 55 |
| CES-D 7 1994 | 3.73 | 4.05 | 1240 | 0 | 21 |
| 19 | - | - | - | - | - |
| Delinquency | 0.27 | 0.45 | 1545 | 0 | 4.87 |
| CES-D 7 1992 | 4.34 | 4.09 | 1069 | 0 | 20 |
| CES-D 20 | 10.08 | 9.36 | 1074 | 0 | 49 |
| CES-D 7 1994 | 3.97 | 4.31 | 1057 | 0 | 21 |
| 20 | - | - | - | - | - |
| Delinquency | 0.24 | 0.42 | 1512 | 0 | 3.87 |
| CES-D 7 1992 | 4.21 | 4.04 | 956 | 0 | 21 |
| CES-D 20 | 9.78 | 9.17 | 961 | 0 | 50 |
| CES-D 7 1994 | 3.82 | 4.25 | 944 | 0 | 21 |
| 21 | - | - | - | - | - |
| Delinquency | 0.23 | 0.43 | 1542 | 0 | 4.40 |
| CES-D 7 1992 | 4.05 | 4.12 | 978 | 0 | 21 |
| CES-D 20 | 9.65 | 9.55 | 987 | 0 | 59 |
| CES-D 7 1994 | 3.74 | 4.08 | 970 | 0 | 21 |
| 22 | - | - | - | - | - |
| Delinquency | 0.20 | 0.34 | 390 | 0 | 2.27 |
| CES-D 7 1992 | 3.79 | 3.89 | 221 | 0 | 20 |
| CES-D 20 | 8.68 | 8.81 | 223 | 0 | 46 |
| CES-D 7 1994 | 3.50 | 4.02 | 217 | 0 | 20 |

## Table 2A

Delinquency and Depression Mean by Minority Status

| **Characteristic** | **Mean** | **SD** | **N** | **Min** | **Max** |
| --- | --- | --- | --- | --- | --- |
| Minority* | - | - | - | - | - |
| Delinquency | 0.27 | 0.45 | 4809 | 0 | 5.63 |
| CES-D 7 1992 | 4.59 | 4.13 | 4424 | 0 | 21 |
| CES-D 20 | 11.01 | 9.53 | 4451 | 0 | 55 |
| CES-D 7 1994 | 4.12 | 4.17 | 4412 | 0 | 21 |
| Nonminority | - | - | - | - | - |
| Delinquency | 0.31 | 0.50 | 7016 | 0 | 5.31 |
| CES-D 7 1992 | 3.81 | 3.90 | 4507 | 0 | 21 |
| CES-D 20 | 8.52 | 8.88 | 4527 | 0 | 59 |
| CES-D 7 1994 | 3.44 | 3.96 | 4463 | 0 | 21 |

*Black or Hispanic

**CESD 7 1992**

# Individual Level

## Table 3A

Individual Level Results: CESD 7 1992 Depression Predicts Delinquency

| **Characteristic** | **Standard Beta** | **Beta** | **t-Statistic** | **95% CI***^1^* | **p-value** |
| --- | --- | --- | --- | --- | --- |
| Intercept | -0.317 | 0.445 | 11.1 | 0.366, 0.523 | <0.001 |
| CESD 7 1992 | 0.101 | 0.012 | 9.60 | 0.009, 0.014 | <0.001 |
| Gender |  |  |  |  |  |
| Female | — | — | — | — | — |
| Male | 0.529 | 0.25 | 25.2 | 0.23, 0.27 | <0.001 |
| Age | -0.094 | -0.020 | -9.02 | -0.024, -0.015 | <0.001 |
| Ethnicity |  |  |  |  |  |
| Minority | — | — | — | — | — |
| NonMinority | 0.109 | 0.052 | 5.24 | 0.032, 0.071 | <0.001 |
| R² = 0.086; F-Statistic = 200; DF1 = 4; DF2 = 8,451; p-value = <0.001; No. Obs. = 8,456  *^1^* CI = Confidence Interval | | | | | |

## Table 4A

Individual Level Results: CESD 7 1992 Delinquency Predicts Depression

| **Characteristic** | **Standard Beta** | **Beta** | **t-Statistic** | **95% CI***^1^* | **p-value** |
| --- | --- | --- | --- | --- | --- |
| Intercept | 0.245 | 5.00 | 14.4 | 4.32, 5.68 | <0.001 |
| Delinquency | 0.107 | 0.908 | 9.60 | 0.723, 1.09 | <0.001 |
| Gender |  |  |  |  |  |
| Female | — | — | — | — | — |
| Male | -0.284 | -1.14 | -12.8 | -1.32, -0.968 | <0.001 |
| Age | -0.003 | -0.005 | -0.272 | -0.043, 0.032 | 0.785 |
| Ethnicity |  |  |  |  |  |
| Minority | — | — | — | — | — |
| NonMinority | -0.206 | -0.829 | -9.63 | -0.998, -0.661 | <0.001 |
| R² = 0.034; F-Statistic = 73.4; DF1 = 4; DF2 = 8,451; p-value = <0.001; No. Obs. = 8,456  *^1^* CI = Confidence Interval | | | | | |

# Between Family Analysis

## Table 5A

Between Family: CESD 7 1992 Depression Predicts Delinquency

| **Characteristic** | **Standard Beta** | **Beta** | **t-Statistic** | **95% CI***^1^* | **p-value** |
| --- | --- | --- | --- | --- | --- |
| Intercept | 0.204 | 0.754 | 9.79 | 0.603, 0.905 | <0.001 |
| CESD 7 1992 Mean | 0.135 | 0.017 | 7.50 | 0.013, 0.022 | <0.001 |
| Gender |  |  |  |  |  |
| Boy/Boy | — | — | — | — | — |
| Girl/Girl | -0.611 | -0.224 | -12.3 | -0.260, -0.189 | <0.001 |
| Mixed Gender | -0.327 | -0.120 | -7.67 | -0.151, -0.089 | <0.001 |
| Age | -0.104 | -0.026 | -5.86 | -0.034, -0.017 | <0.001 |
| Ethnicity |  |  |  |  |  |
| Minority |  | — | — | — | — |
| NonMinority | 0.215 | 0.079 | 5.98 | 0.053, 0.105 | <0.001 |
| R² = 0.080; F-Statistic = 50.5; DF1 = 5; DF2 = 2,917; p-value = <0.001; No. Obs. = 2,923  *^1^* CI = Confidence Interval | | | | | |

## Table 6A

Between Family: CESD 7 1992 Delinquency Predicts Depression

| **Characteristic** | **Standard Beta** | **Beta** | **t-Statistic** | **95% CI***^1^* | **p-value** |
| --- | --- | --- | --- | --- | --- |
| Intercept | 0.010 | 3.88 | 6.33 | 2.68, 5.08 | <0.001 |
| Delinquency Mean | 0.140 | 1.08 | 7.50 | 0.801, 1.37 | <0.001 |
| Gender |  |  |  |  |  |
| BoyBoy | — | — | — | — | — |
| GirlGirl | 0.337 | 0.960 | 6.56 | 0.673, 1.25 | <0.001 |
| Mixed Gender | 0.137 | 0.390 | 3.13 | 0.146, 0.634 | 0.002 |
| Age | -0.004 | -0.008 | -0.224 | -0.076, 0.060 | 0.822 |
| Ethnicity |  |  |  |  |  |
| Minority | — | — | — | — | — |
| NonMinority | -0.326 | -0.930 | -9.00 | -1.13, -0.727 | <0.001 |
| R² = 0.047; F-Statistic = 28.4; DF1 = 5; DF2 = 2,874; p-value = <0.001; No. Obs. = 2,880  *^1^* CI = Confidence Interval | | | | | |

**Discordant Analysis**

## Table 7A

Discordant Results: CESD 7 1992 Depression Predicts Delinquency

| **Characteristic** | **Standard Beta** | **Beta** | **t-Statistic** | **95% CI***^1^* | **p-value** |
| --- | --- | --- | --- | --- | --- |
| Intercept | -0.068 | -0.065 | -0.978 | -0.195, 0.065 | 0.328 |
| Delinquency Mean | 0.783 | 1.02 | 64.8 | 0.986, 1.05 | <0.001 |
| CESD 7 1992 Difference | 0.011 | 0.001 | 0.978 | -0.001, 0.003 | 0.328 |
| CESD 7 1992 Mean | -0.004 | -0.001 | -0.327 | -0.005, 0.003 | 0.744 |
| Age Difference | -0.029 | -0.005 | -2.53 | -0.009, -0.001 | 0.011 |
| Age Mean | 0.014 | 0.004 | 1.20 | -0.003, 0.012 | 0.230 |
| Gender |  |  |  |  |  |
| BoyBoy | — | — | — | — | — |
| GirlGirl | 0.027 | 0.013 | 0.814 | -0.018, 0.044 | 0.415 |
| Mixed Gender | 0.138 | 0.066 | 4.90 | 0.039, 0.092 | <0.001 |
| Ethnicity |  |  |  |  |  |
| Minority | — | — | — | — | — |
| NonMinority | -0.010 | -0.005 | -0.406 | -0.027, 0.017 | 0.68 |
| R² = 0.610; F-Statistic = 569; DF1 = 8; DF2 = 2,914; p-value = <0.001; No. Obs. = 2,923  *^1^* CI = Confidence Interval | | | | | |

## Table 8A

Discordant Results: CESD 7 1992 Delinquency Predicts Depression

| **Characteristic** | **Standard Beta** | **Beta** | **t-Statistic** | **95% CI***^1^* | **p-value** |
| --- | --- | --- | --- | --- | --- |
| Intercept | -0.042 | 0.013 | 0.021 | -1.23, 1.26 | 0.984 |
| Delinquency Mean | 0.003 | 0.031 | 0.206 | -0.265, 0.327 | 0.836 |
| Delinquency Difference | 0.018 | 0.102 | 1.14 | -0.073, 0.278 | 0.252 |
| CESD 7 1992 Mean | 0.567 | 0.687 | 36.1 | 0.649, 0.724 | <0.001 |
| Age Difference | 0.011 | 0.013 | 0.709 | -0.023, 0.049 | 0.479 |
| Age Mean | 0.021 | 0.048 | 1.35 | -0.022, 0.118 | 0.176 |
| Gender |  |  |  |  |  |
| BoyBoy | — | — | — | — | — |
| GirlGirl | -0.005 | -0.016 | -0.105 | -0.313, 0.281 | 0.916 |
| Mixed Gender | 0.010 | 0.035 | 0.276 | -0.216, 0.287 | 0.783 |
| Ethnicity |  |  |  |  |  |
| Minority | — | — | — | — | — |
| NonMinority | 0.078 | 0.271 | 2.51 | 0.060, 0.482 | 0.012 |
| R² = 0.317; F-Statistic = 169; DF1 = 8; DF2 = 2,914; p-value = <0.001; No. Obs. = 2,923  *^1^* CI = Confidence Interval | | | | | |

**CESD 7 1994**

**Individual Level**

## Table 9A

Individual Level Results: CESD 7 1994 Depression Predicts Delinquency

| **Characteristic** | **Standard Beta** | **Beta** | **t-Statistic** | **95% CI***^1^* | **p-value** |
| --- | --- | --- | --- | --- | --- |
| Intercept | -0.312 | 0.473 | 11.7 | 0.394, 0.552 | <0.001 |
| CESD 7 1994 | 0.081 | 0.009 | 7.58 | 0.007, 0.012 | <0.001 |
| Gender |  |  |  |  |  |
| Female | — | — | — | — | — |
| Male | 0.534 | 0.255 | 25.2 | 0.235, 0.275 | <0.001 |
| Age | -0.097 | -0.021 | -9.26 | -0.025, -0.016 | <0.001 |
| Ethnicity |  |  |  |  |  |
| Minority | — | — | — | — | — |
| NonMinority | 0.096 | 0.046 | 4.56 | 0.026, 0.065 | <0.001 |
| R² = 0.083; F-Statistic = 191; DF1 = 4; DF2 = 8,395; p-value = <0.001; No. Obs. = 8,400  *^1^* CI = Confidence Interval | | | | | |

## Table 10A

Individual Level Results: CESD 7 1994 Delinquency Predicts Depression

| **Characteristic** | **Standard Beta** | **Beta** | **t-Statistic** | **95% CI***^1^* | **p-value** |
| --- | --- | --- | --- | --- | --- |
| Intercept | 0.264 | 4.19 | 12.0 | 3.51, 4.88 | <0.001 |
| Delinquency Mean | 0.084 | 0.717 | 7.58 | 0.531, 0.902 | <0.001 |
| Gender |  |  |  |  |  |
| Female | — | — | — | — | — |
| Male | -0.369 | -1.49 | -16.6 | -1.67, -1.32 | <0.001 |
| Age | 0.013 | 0.023 | 1.20 | -0.015, 0.061 | 0.229 |
| Ethnicity |  |  |  |  |  |
| Minority | — | — | — | — | — |
| NonMinority | -0.162 | -0.657 | -7.57 | -0.827, -0.487 | <0.001 |
| R² = 0.039; F-Statistic = 85.7; DF1 = 4; DF2 = 8,395; p-value = <0.001; No. Obs. = 8,400  *^1^* CI = Confidence Interval | | | | | |

# Between Family Analysis

## Table 11A

Between Family: CESD 7 1994 Depression Predicting Delinquency

| **Characteristic** | **Standard Beta** | **Beta** | **t-Statistic** | **95% CI***^1^* | **p-value** |
| --- | --- | --- | --- | --- | --- |
| Intercept | 0.228 | 0.781 | 10.1 | 0.629, 0.933 | <0.001 |
| CESD 7 1994 Mean | 0.131 | 0.016 | 7.23 | 0.012, 0.021 | <0.001 |
| Gender |  |  |  |  |  |
| BoyBoy | — | — | — | — | — |
| GirlGirl | -0.643 | -0.237 | -12.8 | -0.273, -0.201 | <0.001 |
| Mixed Gender | -0.352 | -0.130 | -8.16 | -0.161, -0.099 | <0.001 |
| Age | -0.105 | -0.026 | -5.85 | -0.035, -0.017 | <0.001 |
| Ethnicity |  |  |  |  |  |
| Minority | — | — | — | — | — |
| NonMinority | 0.210 | 0.077 | 5.83 | 0.051, 0.103 | <0.001 |
| R² = 0.081; F-Statistic = 51.0; DF1 = 5; DF2 = 2,874; p-value = <0.001; No. Obs. = 2,880  *^1^* CI = Confidence Interval | | | | | |

## Table 12A

Between Family: CESD 7 1994 Delinquency Predicts Depression

| **Characteristic** | **Standard Beta** | **Beta** | **t-Statistic** | **95% CI***^1^* | **p-value** |
| --- | --- | --- | --- | --- | --- |
| Intercept | -0.132 | 2.55 | 3.97 | 1.29, 3.81 | <0.001 |
| Delinquency Mean | 0.136 | 1.09 | 7.23 | 0.794, 1.38 | <0.001 |
| Gender |  |  |  |  |  |
| BoyBoy | — | — | — | — | — |
| GirlGirl | 0.466 | 1.37 | 8.96 | 1.07, 1.67 | <0.001 |
| Mixed Gender | 0.268 | 0.789 | 6.06 | 0.534, 1.04 | <0.001 |
| Age | 0.009 | 0.018 | 0.499 | -0.053, 0.089 | 0.618 |
| Ethnicity |  |  |  |  |  |
| Minority | — | — | — | — | — |
| NonMinority | -0.233 | -0.686 | -6.36 | -0.898, -0.475 | <0.001 |
| R² = 0.047; F-Statistic = 28.4; DF1 = 5; DF2 = 2,874; p-value = <0.001; No. Obs. = 2,880  *^1^* CI = Confidence Interval | | | | | |

**Discordant Analysis**

## Table 13A

Discordant Results: Differences in CESD 7 1994 Depression Predicts Differences in Delinquency

| **Characteristic** | **Standard Beta** | **Beta** | **t-Statistic** | **95% CI***^1^* | **p-value** |
| --- | --- | --- | --- | --- | --- |
| Intercept | -0.081 | -0.047 | -0.698 | -0.179, 0.085 | 0.485 |
| Delinquency Mean | 0.785 | 1.02 | 64.6 | 0.994, 1.06 | <0.001 |
| CESD 7 1994 Difference | -0.009 | -0.001 | -0.784 | -0.003, 0.001 | 0.433 |
| CESD 7 1994 Mean | -0.016 | -0.003 | -1.31 | -0.006, 0.001 | 0.189 |
| Age Difference | -0.033 | -0.006 | -2.84 | -0.009, -0.002 | 0.005 |
| Age Mean | 0.011 | 0.003 | 0.906 | -0.004, 0.011 | 0.365 |
| Gender |  |  |  |  |  |
| BoyBoy | — | — | — | — | — |
| GirlGirl | 0.038 | 0.018 | 1.13 | -0.013, 0.050 | 0.258 |
| Mixed Gender | 0.149 | 0.072 | 5.22 | 0.045, 0.098 | <0.001 |
| Ethnicity |  |  |  |  |  |
| Minority | — | — | — | — | — |
| NonMinority | -0.002 | -0.001 | -0.069 | -0.023, 0.021 | 0.945 |
| R² = 0.611; F-Statistic = 563; DF1 = 8; DF2 = 2,871; p-value = <0.001; No. Obs. = 2,880  *^1^* CI = Confidence Interval | | | | | |

## Table 14A

Discordant Results: Differences in CESD 7 1994 Delinquency Predicts Differences in Depression

| **Characteristic** | **Standard Beta** | **Beta** | **t-Statistic** | **95% CI***^1^* | **p-value** |
| --- | --- | --- | --- | --- | --- |
| Intercept | -0.028 | 1.12 | 1.78 | -0.113, 2.36 | 0.075 |
| Delinquency Mean | -0.004 | -0.036 | -0.238 | -0.328, 0.256 | 0.812 |
| Delinquency Difference | 0.001 | 0.009 | 0.098 | -0.164, 0.181 | 0.922 |
| CESD 7 1994 Mean | 0.622 | 0.756 | 41.4 | 0.720, 0.792 | <0.001 |
| Age Difference | 0.011 | 0.014 | 0.761 | -0.022, 0.050 | 0.447 |
| Age Mean | -0.004 | -0.011 | -0.304 | -0.080, 0.059 | 0.761 |
| Gender |  |  |  |  |  |
| BoyBoy | — | — | — | — | — |
| GirlGirl | -0.034 | -0.121 | -0.795 | -0.419, 0.177 | 0.427 |
| Mixed Gender | 0.038 | 0.136 | 1.06 | -0.116, 0.387 | 0.291 |
| Ethnicity |  |  |  |  |  |
| Minority | — | — | — | — | — |
| NonMinority | 0.038 | 0.138 | 1.29 | -0.071, 0.347 | 0.196 |
| R² = 0.384; F-Statistic = 223; DF1 = 8; DF2 = 2,871; p-value = <0.001; No. Obs. = 2,880  *^1^* CI = Confidence Interval | | | | | |
